# LncRNA LUCAT1 as a Plasma Biomarker for Assessing Disease Activity in Adult Patients with Crohn's Disease

**DOI:** 10.1155/2021/5557357

**Published:** 2021-09-28

**Authors:** Xiao-yi Kuai, Shun-ying Yu, Xiu-fang Cui, Xiao-jing Zhao, Xia-qiong Mao, Yang Yu, Tong Hu, Hong-jie Zhang, Chun-li Zhou

**Affiliations:** ^1^Department of Gastroenterology, The First Affiliated Hospital of Nanjing Medical University, No. 300 Guangzhou Road, Nanjing, 210029 Jiangsu Province, China; ^2^Department of Gastroenterology, The Affiliated Suzhou Hospital of Nanjing Medical University, No. 242, Guangji Road, Suzhou, 215008 Jiangsu Province, China

## Abstract

**Aim:**

To explore the expression of long noncoding RNA (LncRNA) LUCAT1 in adult patients with Crohn's disease (CD) and evaluate the relationship between LncRNA LUCAT1 and the disease activity in Chinese patients with CD.

**Methods:**

Patients with CD and healthy participants (≥18 years old) were enrolled in this study between January 2018 and December 2019. The expression of LncRNA LUCAT1 in plasma samples was evaluated by quantitative reverse transcription-polymerase chain reaction. Basic characteristics of patients with CD were collected, including gender, age, clinical stage, disease behavior, disease location, C-reactive protein (CRP), platelet (PLT), erythrocyte sedimentation rate (ESR), fecal calprotectin (FC), Crohn's disease activity index (CDAI) score, and simplified Crohn's disease endoscopic score (SES-CD).

**Results:**

In total, 168 patients with CD and 65 healthy participants (≥18 years old) were enrolled in this study. Among them, ninety patients with clinically active CD, seventy-eight patients with CD in clinical remission, forty-eight patients with endoscopically active CD, thirty patients with endoscopically inactive CD, and sixty-five healthy participants. LncRNA LUCAT1 was increased in plasma of patients with CD compared with the control group. The plasma LncRNA LUCAT1 level of patients with CD both in the clinical and endoscopic active phase was higher than that of both the clinical and endoscopic remission phase. The plasma level of LncRNA LUCAT1 in patients with CD was positively correlated with ESR, CRP, FC, CDAI, and SES-CD. There was no significant correlation between the level of LUCAT1 and platelets. The plasma LncRNA LUCAT1 level in patients with CD had significant differences between severe active patients and mild/moderate active patients.

**Conclusion:**

The plasma LncRNA LUCAT1 is positively associated with the disease activity in patients with CD, and it may act as a noninvasive biomarker to identify the degree of disease activity.

## 1. Introduction

Patients with Crohn's disease (CD), a type of inflammatory bowel disease (IBD), suffer from many gastrointestinal symptoms, including diarrhea, abdominal pain, vomiting, and weight loss [1]. These symptoms are nonspecific, and, consequently, the correlation between the symptomatic and the objective measures of disease activity is poor [2].

However, there is currently a lack of effective markers for monitoring IBD disease activity. The current inflammation indicators such as the erythrocyte sedimentation rate (ESR) and C-reactive protein (CRP) do not always express the level of disease activity consistently, and the sensitivity of these tests are usually insufficient to reflect the degree of intestinal inflammation accurately [3]. Therefore, identification of potential biomarkers needed to monitor disease activity for IBD is critical [4].

Previous studies have demonstrated that long noncoding RNAs (LncRNAs) could be considered to act as novel biomarkers for the disease [5]. A previous study reported that there were 438 and 745 differentially expressed LncRNAs in patients with CD, compared with healthy individuals [6]. Therefore, LncRNAs may be the potential diagnostic biomarkers for CD. A recent study by Haberman et al. [7] showed that 15 differentially expressed LncRNAs are differentially expressed in the tissue samples obtained from the patients with CD, such as LINC01272 and LUCAT1. Another study by Wang et al. [8] already had found that LINC01272 was highly expressed in peripheral blood and tissues of IBD, suggesting the potential value of LINC01272 in the diagnosis of IBD. In our previous study, we had verified that LncRNA LUCAT1 was truly increased in tissue samples in patients with CD compared with the control group. Therefore, we focused on plasma LUCAT1 changes in the patients with CD in the study.

In our study, plasma samples from patients with CD and healthy participants were collected. LUCAT1 was measured to determine whether LncRNA LUCAT1 was valuable in assessing and monitoring the disease activity in the patients with CD.

## 2. Materials and Methods

### 2.1. Ethics Statement

The study was approved by the Institutional Review Board and Ethics Committee in the Affiliated Suzhou Hospital of Nanjing Medical University (KL901111). The informed consents were obtained from all participants in this study.

### 2.2. Participants

In this retrospective study, patients with CD and healthy participants (≥18 years old) were enrolled in this study between January 2018 and December 2019 in the Department of Gastroenterology of the Affiliated Suzhou Hospital of Nanjing Medical University. A separate cohort study was conducted to validate the findings of preliminary experiment. The IBD diagnosis was made in accordance with the European Crohn's and Colitis Organization (ECCO) guidelines [9]. The healthy participants were recruited from the geographic region similar to that of the patients in the CD group. These participants were matched by age and sex. They did not have a history of autoimmune or gastrointestinal diseases and were undergoing routine physical examinations at the Health Management Center of the North District that was associated with the Affiliated Suzhou Hospital of Nanjing Medical University.

The inclusion criteria were as follows: (1) diagnosis of CD based on clinical manifestations, radiological findings, and endoscopic and histological criteria; (2) age range from 18 to 75 years old; (3) patients with CD receiving treatment and following up regularly at the Affiliated Suzhou Hospital of Nanjing Medical University; and (4) patients for whom the Montreal classification [10] could be determined. (5) The simplified endoscopic score for Crohn's disease (SES-CD) of the 78 patients with CD was calculated by the physicians experienced in determining the level of disease activity endoscopically. Clinical disease activity was defined as Crohn′s disease activity index (CDAI) points > 150; disease activity on endoscopy was assessed by SES-CD [11], and a score higher than 2 was considered representing active disease on endoscopy [12].

The exclusion criteria were as follows: (1) isolated upper gastrointestinal tract involvement, (2) concurrent autoimmune and gastrointestinal diseases, (3) a history of malignancy or severe infectious disease, and (4) pregnancy or lactation.

### 2.3. Clinical Data and Sample Collection

The clinical characteristics of all the participants, including age, sex, disease type, duration, disease location and behavior, and exposure to CD treatment, were collected from the medical records of the department of gastroenterology. Serum inflammatory biomarkers including C-reactive protein (CRP), erythrocyte sedimentation rate (ESR), and platelet (PLT) count, as well as fecal calprotectin (FC) were tested simultaneously and analyzed. Plasma samples were obtained from the patients with CD and the healthy participants. These samples were centrifuged at 3000 rpm for 10 min and then immediately frozen at -80°C. Plasma samples were stored at a low temperature to avoid their repeated freezing and thawing before the experiment to ensure that when analyzed, all of them would be in the same condition.

### 2.4. Real-Time Quantitative Reverse Transcription-Polymerase Chain Reaction (qRT-PCR)

RNA extraction from the plasma was performed using the QIAGEN RNeasy Serum/Plasma Maxi Kit (QIAGEN, Germany) according to the manufacturer's instructions. Complementary DNA (cDNA) was synthesized using the PrimeScriptTM RT reagent kit (Takara Bio Inc., Otsu, Japan). Quantitative *Reverse Transcription* PCR was performed using the SYBR Premix Ex Taq kit (Takara Bio Inc., Otsu, Japan). The procedure is described briefly as follows: 2 *μ*L cDNA, 5 *μ*L TB Green Premix Ex Taq, 0.2 *μ*L PCR forward primer (10 *μ*M), 0.2 *μ*L PCR reverse primer (10 *μ*M), 0.2 *μ*L ROX reference dye, and 2.4 *μ*L nuclease-free water were added in 200 *μ*L tubes. The reaction mixtures were placed in the Roche 480 system (Roche, Rotkreuz, Switzerland), and the reactions were carried out under the following reaction conditions: 95°C for 30 s, followed by 40 cycles at 95°C for 5 s, and annealing and extension at 60°C for 30 s. Each sample was analyzed in triplicate, and the specificity for each PCR reaction was confirmed by the melt curve analyses. The mRNA expression levels were calculated using the 2^-*ΔΔ*CT^ method with GAPDH as the control [13].The primer sequences used are shown in Table 1.

### 2.5. Statistical Analysis

All data were analyzed using SPSS software (version 20.0; SPSS Inc., Chicago, IL, USA) and GraphPad Prism 8.0 (GraphPad Software Inc., La Jolla, CA, USA). Normally distributed variables were described using mean ± standard deviation; nonnormally distributed variables were presented as median with 25th and 75th percentiles. Comparisons of nonnormally distributed variables were analyzed using the Mann–Whitney *U* test. Correlations were determined using Pearson's or Spearman's coefficient to determine the association between LncRNA LUCAT1 and inflammatory parameters. All *P* values were two-sided, and a *P* value < 0.05 was considered statistically significant.

## 3. Results

### 3.1. Basic Clinical Features of Patients with CD

The basic clinical features of 168 patients with CD are illustrated in Table 2. The study included 90 patients with clinically active CD (average age = 35.9 years, sex ratio male/female = 44/46), 78 patients with CD in clinical remission (average age = 36.7 years, sex ratio male/female = 41/37), and 65 controls (average age = 30.9 years, sex ratio male/female = 35/30) (Table 2). Among the 78 patients with CD in clinical remission, we found 11 patients in endoscopically active CD and 30 patients with endoscopically inactive CD (Table 3). The average expression of LUCAT1 in these 11 patients in endoscopically active CD was 8.0738. The expression of LUCAT1 in average in those 30 patients with endoscopically inactive CD was 3.0452. We found that the differences between these two groups were significant (*P* < 0.001, Table 3).

The colonoscopy was done in 78 patients with CD. Among all these patients, there were 48 patients with endoscopically active CD (average age = 36.5 years, sex ratio male/female = 25/23) and 30 patients with endoscopically inactive CD (average age = 40.5 years, sex ratio male/female = 18/12). Among these 78 patients, we found 37 patients in clinically active CD and 41 patients in clinically inactive CD (Table 3).

### 3.2. The Plasma Levels of LncRNA LUCAT1 in Patients with CD

**Figure 1:**
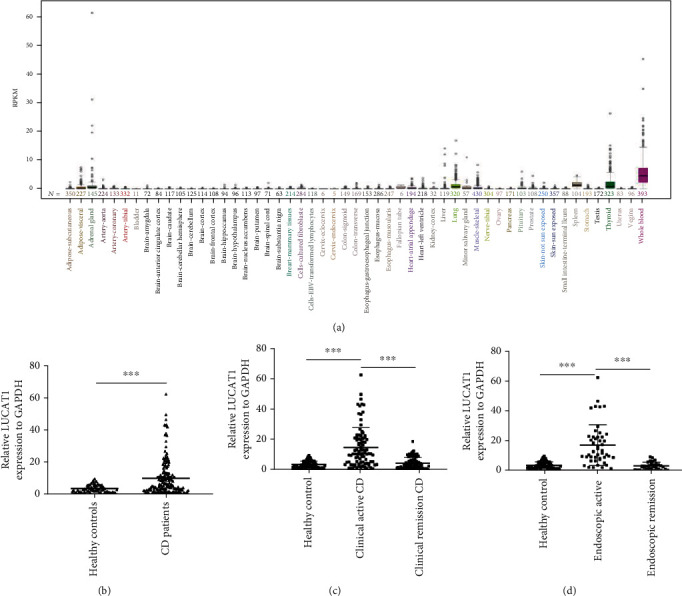
Plasma long noncoding RNA (LncRNA) LUCAT1 expression in the patients with CD. (a) According to the UCSC database, it was predicted that LncRNA LUCAT1 was highly expressed in blood. (b) In the blood samples, LncRNA LUCAT1 was significantly higher in the patients with CD when compared with that in the healthy participants (*P* < 0.001). (c) The expression of LUCAT1 was upregulated in the patients with CD with clinically active disease compared to that of the patients in remission (*P* < 0.001) and the healthy participants (*P* < 0.001). (d) The expression of LUCAT1 was upregulated in the patients with CD with endoscopic evidence of active disease when compared with those in remission observed endoscopically (*P* < 0.001) and the healthy participants (*P* < 0.001). CD: Crohn's disease.

We used the UCSC (University of California Santa Cruz) database (http://genome. ucsc.edu/) to predict the distribution of LUCAT1 expression, and the results indicated that the level of LUCAT1 was higher in the blood than in the colon tissue in the human being (Figure 1(a)). The levels of LUCAT1 in plasma were determined by qRT-PCR. As shown in Figure 1(b), the plasma levels of LUCAT1 were significantly higher in the patients with CD than in the healthy participants (*P* < 0.001). Furthermore, the plasma levels of LUCAT1 in the patients with CD with active disease were higher than the levels in the patients with CD in remission (*P* < 0.001, Figure 1(c)). The plasma levels of LncRNA LUCAT1 in the patients with endoscopically active CD according to the endoscopy were significantly higher than the levels in those patients that showed remission endoscopically (*P* < 0.001) (Figure 1(d)). There was no significant difference in the plasma levels of LUCAT1 between the patients with CD in clinical remission and the healthy participants. Similarly, the plasma LUCAT1 levels in the patients with CD in endoscopic remission were comparable to those in the normal participants.

### 3.3. Association of LUCAT1 with the Inflammatory Biomarkers and Disease Activity in Patients with CD

**Figure 2:**
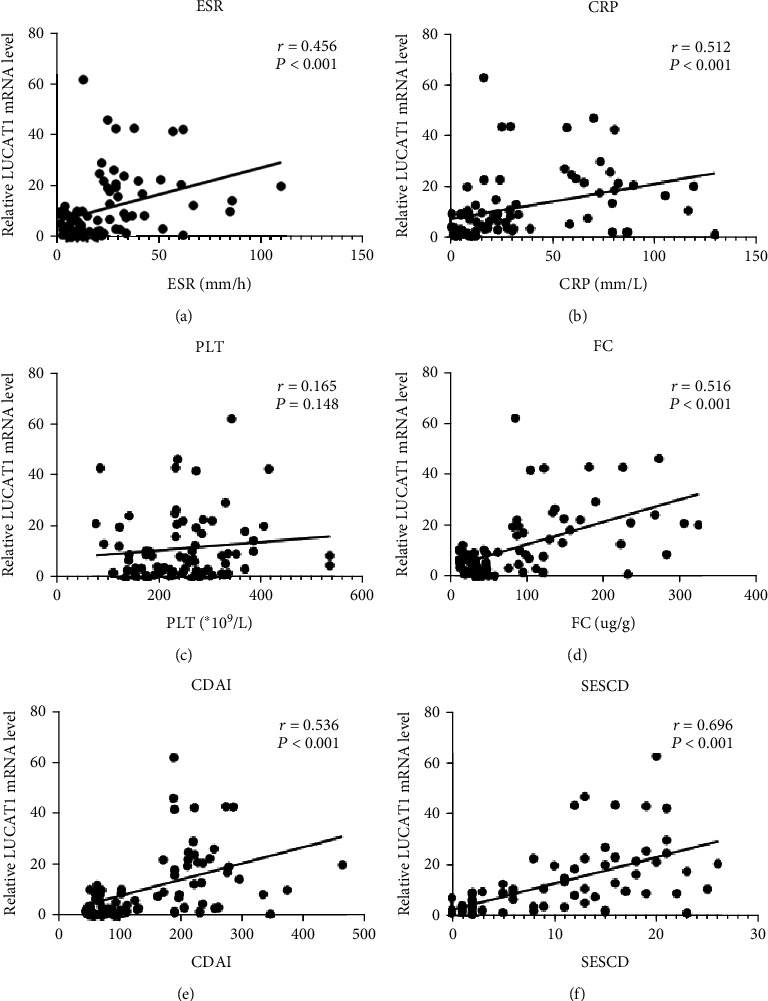
Correlation of LUCAT1 with CDAI, ESR, CRP, FC, PLT, and SES-CD in the patients with CD. (a) Correlation analysis of LUCAT1 expression with ESR. (b) Correlation analysis of LUCAT1 expression with CRP. (c) Correlation analysis of LUCAT1 expression with PLT. (d) Correlation analysis of LUCAT1 expression with FC. (e) Correlation analysis of LUCAT1 expression with CDAI. (f) Correlation analysis of LUCAT1 expression with SES-CD. *P* < 0.05 was considered statistically significant. CD: Crohn's disease; CDAI: Crohn's disease activity index; CRP: C-reactive protein; ESR: erythrocyte sedimentation rate; FC: fecal calprotectin; PLT: platelet.

To assess the relationship between LUCAT1 and the disease activity in the patients with CD, we analyzed the association between the level of LUCAT1 and the inflammatory biomarkers such as ESR, CRP, PLT, and FC. As shown in Figures 2(a) and 2(b), the plasma level of LUCAT1 had a moderately significant positive correlation with ESR (*r* = 0.456, *P* < 0.001) and CRP (*r* = 0.512, *P* < 0.001) in the patients with CD. However, no significant correlation was found between LUCAT1 and PLT counts (*r* = 0.112, *P* = 0.105; Figure 2(c)). Additionally, the levels of LUCAT1 were moderately positively correlated with FC (*r* = 0.516, *P* < 0.001; Figure 2(d)). The results showed that LUCAT1 was positively associated with CDAI (*r* = 0.536, *P* < 0.001; Figure 2(e)). Furthermore, the level of LUCAT1 also had a significantly positive correlation with SES-CD in patients with CD (*r* = 0.696, *P* < 0.001; Figure 2(f)).

### 3.4. Association of LncRNA LUCAT1 with Clinical Parameters in Patients with CD

**Figure 3:**
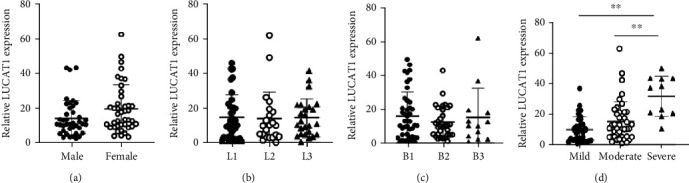
Correlation of the plasma LncRNA LUCAT1 and clinical parameters in the patients with CD. (a) The relative expression of LUCAT1 in male/female patients. (b) The relative expression of LUCAT1 in patients related to the location at L1/L2/L3. (c) The relative expression of LUCAT1 in in patients related to the disease behaviors at B1/B2/B3. (d) The relative expression of LUCAT1 in patients with mild/moderate/severe CD and in controls. ^∗∗^*P* < 0.01.

We analyzed the associations between LncRNA LUCAT1 and clinical parameters. There was a significant difference in this respect was noted between patients in severe stage and patients in mild/moderate stages (Figure 3(d)). On the contrary, LUCAT1 level showed no difference in gender, disease location, and behavior (*P* > 0.05) (Figures 3(a)–3(c)).

### 3.5. Validation of the Association of LncRNA LUCAT1 with Specific Inflammatory Biomarkers and Disease Activity in a Separate Cohort

**Figure 4:**
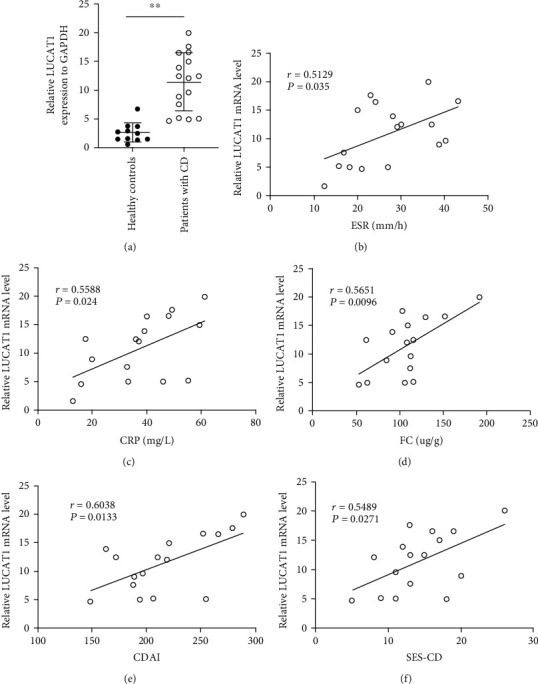
Association of LncRNA LUCAT1 with the inflammatory biomarkers and disease activity in the separate cohort. (a) LncRNA LUCAT1 was significantly higher in the patients with CD when compared with that in the healthy participants (*P* < 0.01) (b) Correlation analysis of LUCAT1 expression with ESR. (c) Correlation analysis of LUCAT1 expression with CRP. (d) Correlation analysis of LUCAT1 expression with FC. (e) Correlation analysis of LUCAT1 expression with CDAI. (f) Correlation analysis of LUCAT1 expression with SES-CD. *P* < 0.05 was considered statistically significant. CD: Crohn's disease; CDAI: Crohn's disease activity index; CRP: C-reactive protein; ESR: erythrocyte sedimentation rate; FC: fecal calprotectin; PLT: platelet.

To validate our findings, an independent patient cohort was conducted, which included 16 patients with CD and 10 healthy controls. The clinical information of the 16 participants in the separate cohort is illustrated in Table 4. Plasma LUCAT1 was determined by qRT-PCR. As shown in Figure 4(a), the plasma LUCAT1 was still significantly higher in the patients with CD than that in the health participants (*P* < 0.01).

To assess the relationship between LUCAT1 and the disease, we also analyzed the association between the LUCAT1 and the inflammatory biomarkers such as ESR, CRP, and FC. As shown in Figures 4(b) and 4(c), the plasma LUCAT1 had a moderately significant positive correlation with ESR (*r* = 0.5129, *P* = 0.035) and CRP (*r* = 0.5588, *P* = 0.024) in the patients with CD. Additionally, the level of LUCAT1 was also moderately positively correlated with FC (*r* = 0.5651, *P* = 0.0096; Figure 4(d)). The results showed that LUCAT1 was positively associated with CDAI (*r* = 0.6038, *P* = 0.0133; Figure 4(e)). Furthermore, the level of LUCAT1 also had a significantly positive correlation with SES-CD in patients with CD (*r* = 0.5489, *P* = 0.0271; Figure 4(f)).

## 4. Discussion

We investigated the alterations in LUCAT1 levels in patients with CD. The results showed higher plasma LUCAT1 levels in patients with CD when compared with healthy controls, and the LUCAT1 levels were positively correlated with the disease activity.

Recently, there has been an increasing focus upon noncoding RNA (ncRNA) to determine the potential pathogenic mechanism of IBD. As a new group of ncRNAs, the increasing evidence confirmed that LncRNAs were involved in the pathogenesis of inflammation [14]. For example, LncRNA-Cox2 has been reported to be a downstream target of Toll-like receptor (TLR) signaling and can act as a transcriptional cofactor by interacting with various regulatory complexes [15]. In addition, research has shown that LncRNA is also involved in inflammation [16] and has been shown to regulate intestinal epithelial barrier function [17]. LncRNAs were also considered novel biomarkers for the diagnosis of solid cancers.

Recently, a study by Yarani et al. [18] showed that there are 12 differentially expressed LncRNAs associated with CD, including LncRNA LUCAT1. In our previous study, we had verified that the expression of LncRNA LUCAT1 increased in the patients with CD. We further searched the UCSC databases where it was predicted that the distribution and expression of LUCAT1 in the blood might also be very high. Therefore, we hypothesized that the expression of LUCAT1 in the blood of the patients with CD would also increase as it can act as a marker of inflammation. To verify this hypothesis, we determined the levels of LncRNA LUCAT1 by performing real-time PCR on the blood samples of the patients with CD. The results showed that the plasma levels of LUCAT1 in the patients with CD were significantly higher than the levels in the control group. In addition, the plasma levels of LncRNA LUCAT1 in the patients with active disease (CDAI points > 150, SES‐CD > 2) were higher than those in the patients with CD in remission (*P* < 0.001, CDAI points ≤ 150, SES‐CD ≤ 2). The level of LncRNA LUCAT1 was positively correlated with the disease activity indices such as FC, ESR, SES-CD, and CDAI in the patients with CD. LUCAT1 levels can be used to differentiate between patients with severe stage and patients with mild/moderate. Taking these results together, it seems likely that increased inflammatory responses tend to associate with higher LUCAT1 levels in patients with CD with higher CDAI.

There are several limitations to this study. Firstly, the sample size was insufficient, which may have affected the results. Secondly, the applicable conclusions need to be derived from more diverse disease control groups. Finally, we did not further explore the specific pathogenic mechanism of LUCAT1 in patients with CD.

## 5. Conclusion

In summary, the results from this study confirm that patients with active CD have higher LUCAT1 expression. Specifically, these findings indicate that higher LUCAT1 levels tend to associate with increased inflammatory responses in patients with CD. Based on these data, we raise the question whether the expression of LncRNA LUCAT1 might act as a pathogenic factor in CD. Nevertheless, these findings still need to be supported by further investigation to assess the exact molecular mechanism of LUCAT1 in patients with CD.

## Figures and Tables

**Table 1:** Primers used in qPCR for validation.

Target	Primers	
LUCAT1	Sense	5′-CGTGAATGATAGTGAGGACTC-3′
	Antisense	5′-TGAACGATTTGCCACACAAGGA-3′
GAPDH	Sense	5′-GGCACAGTCAAGGCTGAGAATG-3′
	Antisense	5′-ATGGTGGTGAAGACGCCAGTA-3′

**Table 2:** Clinical characteristics of Crohn's disease and healthy control.

	Crohn's disease				Healthy control
	Clinical active	Clinical remission	Endoscopic active	Endoscopic remission	
Number (*n*)	90	78	48	30	65
Age (years, median (min, max))	35.9 (15-82)	36.7 (15-78)	36.5 (15-68)	40.5 (25-60)	30.9 (22-62)
Gender (M/F) (*n*)	44/46	41/37	25/23	18/12	35/30
Disease duration (months, median (min, max))	120(12-372)	98(12-360)	108(11-273)	132(13-360)	
Current therapy (*n*)					
5-Aminosalicylates	44	54	26	20	
Immunosuppressants	30	38	12	15	
Biologics	68	61	37	22	
Nutritional therapy	17	10	9	2	
Disease location					
L1	43	20	22	10	
L2	22	16	11	6	
L3	25	42	15	14	
L4	0	0	0	0	
Disease behaviors					
B1	44	28	25	13	
B2	33	38	16	12	
B3	13	12	7	5	
ESR (mm/h, median (min, max))	31.1 (7-110)	8 (2-22)	29 (2-110)	9.25 (2-22)	NA
CRP (mg/L, median (min, max))	44.5 (8.1-155.1)	8.5 (0.23-31.1)	33.7 (0.7-129)	9.1 (0.2-30.7)	NA
PLT ((×10^9^/L, median (min, max))	286.5 (78.2-535)	191.8 (112-392)	271.7 (78.2-535)	199.5 (112-331)	NA
FC ((*μ*g/g, median (min, max))	132.3 (39.8-323.2)	32.3 (8.7-52.2)	100.7 (10.7-323.2)	33.4 (13.2-58.2)	NA
CDAI (median (min, max))	223.5 (1533.5-424.2)	69.0 (45.3-141.8)	208.8 (54.8-424.2)	71.2 (43.3-96.7)	NA

CD: Crohn's disease; CDAI: clinical disease activity index; CRP: C-reactive protein; ESR: erythrocyte sedimentation rate; FC: fecal calprotectin; PLT: platelet; F: female; M: male; NA: not available; min: minimum; max: maximum; L1: ileal; L2: colonic; L3: ileocolonic; L4: upper; B1: nonstricturing, nonpenetrating; B2: stricturing; B3: penetrating.

**Table 3:** Expression of LUCAT1 in average among the different groups.

	Endoscopic active CD (*n*)	Expression of LUCAT1 in average	Endoscopic remission CD (*n*)	Expression of LUCAT1 in average	*P* value
Clinical active CD (*n*)	37	19.3768	0	NA	
Clinical remission CD (*n*)	11	8.0738	30	3.0452	*P* < 0.01

**Table 4:** Clinical information of the participants in the separate cohort.

	CD (*n* = 16)	Control (*n* = 10)
Age (y)	28.9 (18–32)	27.6 (18–34)
Sex (M/F) (*n*)	9/7	5/5
ESR (mm/h)	23.21 (12.39–43.12)	NA
CRP (mg/L)	31.49 (12.68–61.24)	NA
FC	82.32 (53.26–192.17)	NA
CDAI (scores)	195.95 (148.53–289.17)	NA
SES-CD (scores)	14.12 (5-26)	NA

CD: Crohn's disease; CDAI: clinical disease activity index; CRP: C-reactive protein; ESR: erythrocyte sedimentation rate; FC: fecal calprotectin; PLT: platelet; F: female; M: male; NA: not available.

## Data Availability

The data used to support the findings of the study are available from the corresponding author on reasonable request.
